# Limited Nosocomial Transmission of Drug-Resistant Tuberculosis, Moldova

**DOI:** 10.3201/eid2905.230035

**Published:** 2023-05

**Authors:** Ecaterina Noroc, Dumitru Chesov, Matthias Merker, Matthias I. Gröschel, Ivan Barilar, Viola Dreyer, Nelly Ciobanu, Maja Reimann, Valeriu Crudu, Christoph Lange

**Affiliations:** Chiril Draganiuc Phthisiopneumology Institute, Chisinau, Moldova (E. Noroc, N. Ciobanu, V. Crudu);; Nicolae Testemitanu State University of Medicine and Pharmacy, Chisinau (D. Chesov);; Research Center Borstel, Borstel, Germany (D. Chesov, M. Merker, I. Barilar, V. Dreyer, M. Reimann, C. Lange);; German Center for Infection Research (DZIF), Borstel (M. Merker, I. Barilar, V. Dreyer, M. Reimann, C. Lange);; Harvard Medical School, Boston, Massachusetts, USA (M.I. Gröschel);; Charité–Universitätsmedizin Berlin, Berlin, Germany (M.I. Gröschel);; University of Lübeck, Lübeck, Germany (M. Reimann, C. Lange);; Baylor College of Medicine and Texas Children’s Hospital, Houston, Texas, USA (C. Lange)

**Keywords:** tuberculosis and other mycobacteria, multidrug-resistant tuberculosis, rifampin-resistant tuberculosis, TB, Moldova, WGS, Ural, Beijing, antimicrobial resistance, bacteria

## Abstract

Applying whole-genome-sequencing, we aimed to detect transmission events of multidrug-resistant/rifampin-resistant strains of *Mycobacterium tuberculosis* complex at a tuberculosis hospital in Chisinau, Moldova. We recorded ward, room, and bed information for each patient and monitored in-hospital transfers over 1 year. Detailed molecular and patient surveillance revealed only 2 nosocomial transmission events.

The main factor driving the epidemic of multidrug-resistant (MDR) and rifampin-resistant (RR) tuberculosis (TB) in Eastern Europe is active transmission of drug-resistant *Mycobacterium tuberculosis* complex (MTBC) (*1*). The role of nosocomial transmission of drug-resistant MTBC during prolonged hospitalizations remains poorly understood (*2*,*3*). We prospectively aimed to detect nosocomial transmission events at a TB referral hospital in Chisinau, the capital of Moldova.

## The Study

**Figure 1:**
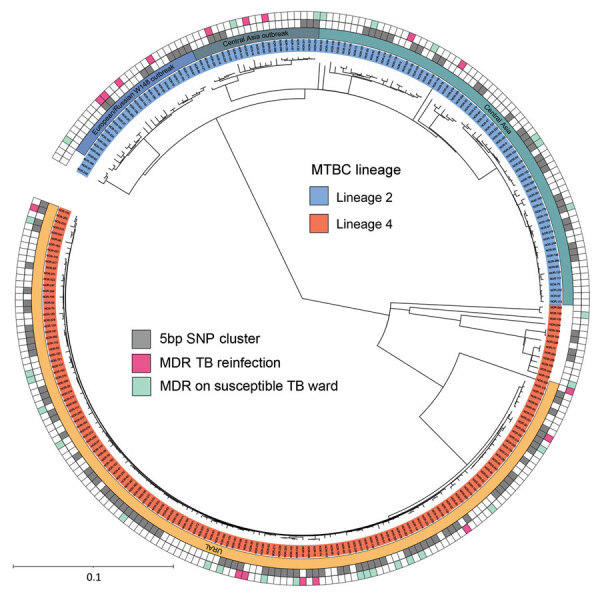
Maximum-likelihood phylogeny of 268 MDR TB isolates in study of limited nosocomial transmission of drug-resistant tuberculosis, Moldova. In outside circle, gray squares represent patient isolates with a maximum genetic distance of 5 single-nucleotide polymorphisms as a surrogate for recent transmission; pink squares indicate focus patients who acquired a new MDR MTBC strain during earlier treatment for drug-susceptible TB. Scale bar indicates number of substitutions per site. MDR, multidrug-resistant; MTBC, *Mycobacterium tuberculosis* complex; SNP, single-nucleotide polymorphism; TB, tuberculosis.

We performed the study at the Chiril Draganiuc Phthisiopneumology Institute, Chisinau, Moldova. From July 1, 2014, through June 30, 2015, we prospectively tracked patients’ locations by room within the hospital, on the basis of the beds patients occupied each day during their hospital stays. We evaluated sputum samples by mycobacterial culture and performed phenotypic drug-susceptibility testing for all MTBC strains at admission. Sputum cultures for growth of MTBC were performed at least at the end of the second month, the fifth month, and the end of treatment in patients with drug-susceptible TB; for patients with MDR/RR TB, cultures were performed on a monthly basis until no growth of MTBC was detectable and quarterly thereafter (*4*). MTBC strains resistant to isoniazid and rifampin underwent whole-genome sequencing for genotypic prediction of drug resistance and phylogenetic comparison. All patients admitted to the study were followed up for 2 years after enrollment (Appendix 1). In total, 2,490 patients were admitted during the study period (Table 1; Appendix 1 Figure 1). The study was approved by the Research Ethical Committee of the State University of Medicine and Pharmacy (#15_49/2014), Chisinau, Moldova.

**Table 1:** Characteristics of patients by hospital departments in study of limited nosocomial transmission of drug-resistant TB, Moldova*

Characteristic	Total, n = 2,490	Dept 1, n = 401	Dept 2, n = 445	Dept 3, n = 1,127	MDR TB dept, n = 156	EP-TB and Surgery dept, n = 361
Mean age, y (+SD)	50.9 (+17.4)	43.7 (+13.8)	45.6 (+12.1)	59.4 (+17.5)	37.2 (+12.7)	45.2 (+15.4)
Sex						
M	1,623 (65.2)	288 (71.8)	356 (80.0)	609 (54.0)	110 (70.5)	260 (72.0)
F	867 (34.8)	113 (28.2)	89 (20.0)	518 (46.0)	46 (29.5)	101 (28.0)
TB	1,379 (55.4)	397 (99)	442 (99.3)	57 (5,1)	156 (100)	327 (90.6)
Culture positive TB	938 (68.0)	296 (74.6)	331 (74.9)	25 (43.9)	142 (91)	144 (44.0)
Culture negative or missed TB, Xpert positive	78 (5.7)	20 (5.0)	27 (6.1)	8 (14.0)	9 (5.8)	14 (4.3)
Patients without microbiological confirmation of TB	363 (26.3)	81 (20.4)	84 (19.5)	24 (42.9)	5 (3.2)	169 (51.7)
MDR TB by culture	307 (31.4)	39 (11.5)	77 (21.3)	7 (25)	141 (99.3)	43 (29.2)
New TB cases	1,034 (75)	380 (95.7)	259 (58.6)	52 (91.2)	82 (52.6)	261 (79.8)
Relapse TB	233 (16.9)	16 (4.0)	135 (30.5)	5 (8.9)	30 (19.2)	47 (14.4)
Retreatment after LTF	50 (3.6)	1 (0.3)	23 (5.2)	0	15 (9.6)	11 (3.4)
Retreatment after failure	62 (4.5)	0	25 (5.7)	0	29 (18.6)	8 (2.5)
Pulmonary TB	1,114 (80.8)	369 (92.9)	425 (96.2)	44 (77.2)	149 (95.5)	127 (38.8)
Extrapulmonary TB	183 (13.3)	8 (2)	3 (0.7)	11 (19.6)	1 (0.6)	160 (49)
Pulmonary and extrapulmonary TB	82 (5.9)	20 (5.5)	14 (3.2)	2 (3.6)	6 (3.9)	40 (12.2)

*Values are no. (%) except as indicated. Xpert, Xpert MTB/RIF assay (Cepheid, https://www.cepheid.com). Dept, department; EP-TB, extrapulmonary TB; LTF, loss to follow-up; MDR, multidrug-resistant; TB, tuberculosis.

The number of patients with a confirmed diagnosis of TB by culture or the Xpert MTB/RIF assay (Cepheid, https://www.cepheid.com) was 1,016/1,379 (73.7%) (Table 1). Drug-susceptible strains of MTBC were found in 567/938 patients (60.5%), strains of mono/polydrug-resistant MTBC in 64/938 patients (6.8%), and strains of MDR/RR MTBC in 307/938 (32.7%) patients with detectable MTBC in culture. A total of 297/307 (96.7%) MDR/RR strains were available for analysis.

**Figure 2:**
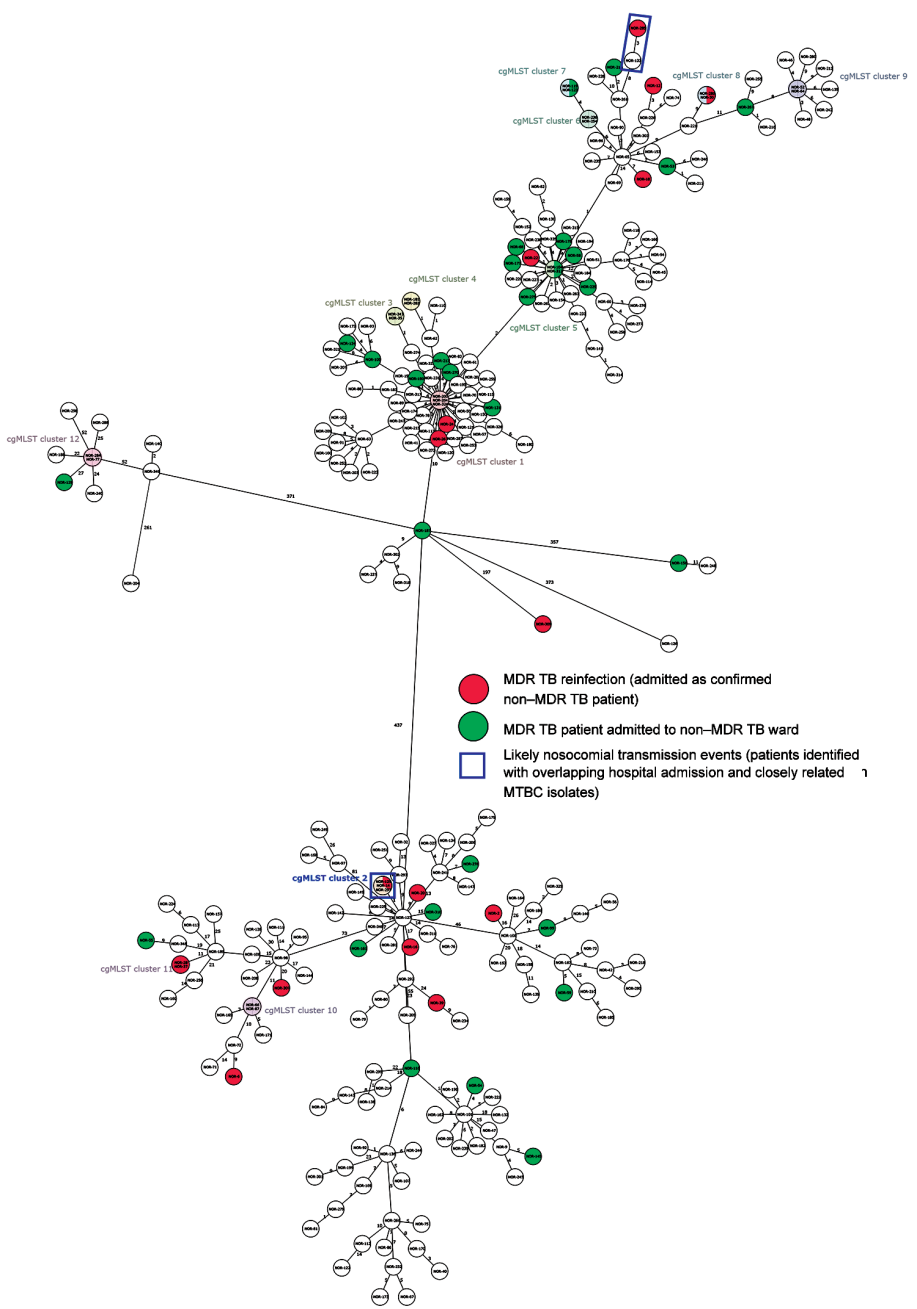
Phylogenetic network representing the genomic relatedness of all patient isolates in study of limited nosocomial transmission of drug-resistant tuberculosis, Moldova. Network is based on a core genome multilocus sequence type analysis. Pink indicates patients who acquired MDR TB, green indicates MDR TB patients initially admitted to a non–MDR TB ward; blue boxes indicate identified likely nosocomial transmission events. MDR, multidrug-resistant; MTBC, *Mycobacterium tuberculosis* complex; TB, tuberculosis.

The median length of hospital stay was 22 days (interquartile range [IQR] 9–62 days) (Appendix 1 Figure 2, panel A). After admission, 75 patients were transferred to a different department than the one in which they were initially hospitalized (Appendix 1 Figure 2, panel B). Median length of stay until transfer to another department was 7 days (IQR 4–19 days) (Appendix 1 Figure 2, panel C).

A total of 41 patients with MDR/RR TB were initially admitted to a non–MDR TB departments. Of those, 33 patients were later transferred to the MDR TB department, and 8 patients were transferred to a different non–MDR TB department (Appendix 1 Figure 3, panel A). The median duration of stay for patients with MDR/RR TB in non–MDR TB departments was 7 days (IQR 4–18 days), and cumulative duration of stay was 631 days (Appendix 1 Figure 3, panel B). The median number of room-sharing contacts of patients with MDR/RR TB on non–MDR TB wards was 3 patients (IQR 2–5 patients), and the cumulative number of patients was 144.

A total of 17 patients (focus patients) with drug-susceptible MTBC strains at enrollment were found to be reinfected with an MDR/RR MTBC strain on follow-up. Only 1/144 roommates of the 41 patients with MDR/RR TB initially admitted to a non–MDR TB ward was in the same room with 1 of the 17 focus patients who potentially acquired MDR/RR TB on the non–MDR TB ward. For 297 patients with culture-confirmed MDR/RR TB at study enrollment and 17 focus patients with an MDR/RR MTBC reinfection detected during the follow-up period, 268 next-generation sequencing datasets were available for the molecular epidemiologic analysis (including all 17 strains from the focus patients).

The bacterial population consisted of MTBC lineage 2 isolates (116/268, 43.3%) and lineage 4 isolates (152/268, 56.7%). Isolates in lineage 2 were typed as Central Asia L2-sublineage (67/116, 57.8%), Central Asia outbreak (21/116, 18.1%), and Europe/Russia W148 outbreak (25/116, 21.6%), whereas lineage 4 almost exclusively consisted of sublineage 4.2.1, URAL genotype (139/152, 91.4%) (Figure 1). We used sequence data to predict the resistance phenotype on the basis of direct association with previously described resistance-conferring single-nucleotide polymorphisms (SNPs) (Appendix 1 Figure 4).

To highlight putative transmission events between the focus patients and concurrently admitted patients with MDR/RR TB, we performed a molecular cluster analysis on the basis of pairwise genetic distance between all isolates (Figure 2). Overall, 124/268 (46.3%) patients were part of 1 of the 28 identified clusters, including 7/17 focus patients (Appendix 2 Table). Only 2/17 focus patients (sample ID 14 and ID 285) had a possible direct link in the hospital with a difference of <6 SNPs between the infecting MTBC strains, as well as 13 days and 29 days overlap in the hospital with their putative index case (Table 2; Appendix 1).

**Table 2:** Likely nosocomial transmission events of drug-resistant TB, Moldova*

Patient with acquired MDR TB	Possible index cases for transmission	SNP difference	Days overlap in the hospital	Days overlap on the same ward	Days overlap in the same room	Strain
NOR-14	NOR-297	0	13†	0	0	2.2.1 Central Asia outbreak
NOR-285	NOR-133	3	29	27	23	4.2.1 URAL

*MDR TB, multidrug-resistant tuberculosis; SNP, single-nucleotide polymorphism; TB, tuberculosis. †Patients had already shared 52 d in the hospital before the official start of the study.

## Conclusions

Nosocomial transmission of MTBC infection in high-burden settings has been reported previously (*5*–*7*). We prospectively aimed to detect transmission events of MDR/RR strains of MTBC at the TB referral center in Moldova, a country of high MDR/RR TB incidence. By matching each patient with a specific ward, room, and bed in the hospital for each day of the year, we were able to identify which of 307 patients with MDR/RR TB were initially wrongly allocated to a non–MDR TB ward, potentially leading to nosocomial transmission to other patients. Forty-one patients with MDR/RR TB initially spent a total of 631 days on non–MDR TB wards before drug-resistant TB was identified and they were transferred to an MDR TB ward. By using whole-genome sequencing on MDR/RR strains of MTBC from putative index patients and patients with drug-susceptible TB in whom MDR/RR TB then developed during follow-up, we identified only 2 highly likely transmission events, indicating a low rate of nosocomial transmission of MDR/RR strains of MTBC. Systematic implementation of basic infection control measures at the Chiril Draganiuc Phthisiopneumology Institute after previous indications of nosocomial transmission of MTBC (*3*) might have been effective in reducing TB transmission (*8*). However, these findings are limited by the high clonality of MTBC strains in patients with MDR/RR TB in Moldova (*9*), where more than one third of all incident TB cases are affected by multidrug/rifampin resistance (*10*). Our results call for further community efforts to reduce transmission of drug-resistant TB.

The first limitation of this study is that isolates were sampled only in the hospital, which could have introduced selection bias with persons without access to healthcare. However, because TB care in Moldova is centralized and provided free of charge, the effect of those factors should be minimal. Second, patients admitted to the hospital might have been subsequently readmitted to another hospital, in which case transmission events would have been missed. However, use of the national TB reporting database for follow-up minimizes the potential effect of this limitation. Third, the short time frame of this study might have missed transmission events, although most cases occur within 1 year of infection. Fourth, although a diagnostic delay occurred, transmission of MDR/RR MTBC could have been reduced already if patients had received empiric partly active treatment regimens. Finally, the rate of detected transmission might have been higher if transmission from patients with MDR/RR TB to other patients with MDR/RR TB had also been assessed.

In summary, in a detailed prospective evaluation at the TB referral hospital in Chisinau, Moldova, a high burden country of drug-resistant TB, we found that the rate of nosocomial transmission of MDR/RR strains of MTBC is low. Our results indicate the need for further community efforts to reduce transmission of drug-resistant strains of MTBC in high-burden settings.

Appendix 1 Additional information about limited nosocomial transmission of drug-resistant tuberculosis, Moldova.

Appendix 2 Additional data on limited nosocomial transmission of drug-resistant tuberculosis, Moldova.
