# Supplementary material for: Limited Nosocomial Transmission of Drug-Resistant Tuberculosis, Moldova
Source: Emerg Infect Dis. 2023 May;29(5):1046–50. doi: 10.3201/eid2905.230035 (PMC10124655; doi:10.3201/eid2905.230035)
Supplement: Appendix 1 — Additional information about limited nosocomial transmission of drug-resistant tuberculosis, Moldova. [file 23-0035-Techapp-s1.pdf]

Article DOI: https://doi.org/10.3201/eid2905.230035

EID cannot ensure accessibility for supplementary materials supplied by authors. Readers who have difficulty accessing supplementary content should contact the authors for assistance.

Limited Nosocomial Transmission of Drug-Resistant Tuberculosis, Moldova

Appendix 1

Supplementary Methods

Settings

Chiril Draganiuc Phthisiopneumology Institute is a tertiary hospital, located in Chisinau, the capital of the Republic of Moldova. The institute is specialized in the diagnosis and treatment of all forms of TB as well of non-TB pulmonary diseases in adult patients. At the time of study enrollment, the institute included 4 main in-patient departments: Phthisiopneumology #1 (PP1) with 55 beds in 10 rooms, Phthisiopneumology #2 (PP2) with 80 beds in 14 rooms, Phthisiopneumology #3 (PP3) with 50 beds in 11 rooms, Multidrug resistant TB department (MDRd) with 50 beds in 16 rooms and Extrapulmonary TB and Surgery ward (ETBS) with 60 beds in 20 rooms, as well there is an intensive care unit (ICU) for 5 beds. Patients are admitted to different wards according to the type of the disease and resistance profile of the Mtbc. To the PP1 are admitted patients with new susceptible respiratory TB, to the PP2 - retreatment cases of susceptible respiratory TB, to the PP3 mainly patients with non-TB pulmonary disease and those with limited microbiological negative respiratory TB, to the MDRd - cases of MDR or rifampin resistant TB, and to the ETBS cases of extrapulmonary TB or those with pulmonary TB that require surgery treatment.

During the hospital admission the patients could be transferred between the wards. This usually occur when a patient admitted to a non-TB ward is diagnosed with TB or when in a TB patient a resistance pattern different of the initial pattern is documented. As well the transfer of the patients between the wards could occur if surgery or intensive care is required.

During their stay in the hospital the patients are allowed to go outside for walk or smoking in the hospital yard where they interact with patients from other wards. As well, the patients share the elevator and use the same staircases. Also, the patients can come in contact in the hospital canteen, where are allowed only smear negative patients from PP1 and PP2. To mention that smear positive TB patient a not allowed to go to the canteen, they receive their food at a distribution point in their ward and consume it in their own rooms. All patients from MDRd and PP3 received the food exclusively at the distribution point in their department. Another possibility for a close contact between the patients from the same ward is the daily picking of the pills and injections at the nurse desk in the ward.

During their movement outside their rooms within the hospital all the patients are demanded to wear a surgical mask as well during their contact with the medical personnel. Other infection control measures routinely applied are wearing of the respirators by the personnel, use of the UV lamp for disinfection, limited access of the patients to the personnel area, natural ventilation of the rooms (limited during the wintertime).

Treatment policy

Tuberculosis treatment in the Republic of Moldova is offered free of charge and it is guide by the National Clinical Protocol - Tuberculosis in adults which is accordance with the WHO treatment guidelines [10].

Patients with susceptible TB receive a standard treatment with 4 drugs (isoniazid, rifampin, pyrazinamide, ethambutol) during the first 2–3 months (dependent of the achievement of the sputum culture conversion) followed by 4 months of isoniazid and rifampin. In most of the case the total treatment duration for susceptible TB is 6 months but could be prolonged in some cases.

During the study period, patients with MDR-TB initially started treatment with a standardized regimen of five 2nd-line TB drugs including a fluroquinolone (levofloxacin or moxifloxacin), a 2nd-line injectable (capreomycin or amikacin), ethionamide, cycloserine and pyrazinamide) which was adjusted, when necessary, once results of phenotypic or molecular drug susceptibility testing (DST) became available. The treatment duration in MDR TB patients is linked to the time of sputum culture conversion and consist of an initial 6–8 months of intensive phase followed by a continuation phase with a duration of 12–16 months.

Usually in patients with smear positive susceptible TB, particularly in those with advanced disease, as well in MDR-TB patients treatment is initiated in TB hospital, where the patients are admitted at least until the smear sputum conversion is achieved and the final treatment scheme is decided. The treatment during hospital admission is supervised by a pneumologist specialized in treatment of TB patients (phthisio-pneumologist). After hospital discharge the patient continues to receive his treatment in outpatient conditions. In outpatient the treatment is provided by the patient's family doctor or phthisio-pneumologist who has his office closest to the patient domicile. The patient must come to the medical office daily to receive his medication under direct observation.

Microbiological methods

The standard battery of microbiological tests performed on admission for the detection of Mtbc performed in all patients hospitalized in the Chiril Draganiuc Institute are sputum smear microscopy for acid fast bacilli (AFB), GeneXpert Mtbc/Rif (Cepheild, Sunnyvale, USA), and a sputum culture for Mtbc on BACTEC MGIT 960 (Becton Dickinson, USA) and on Lowenstein-Jenses (LJ) media. In case of a positive sputum culture an initial culture-based drug susceptibility test (pDST) for isoniazid, rifampin ethambutol and in some patients also for pyrazinamide is performed. Additionally, if pDST detect a MDR resistant pattern or rifampin resistance is detected by GeneXpert, a pDST for latest generation fluoroquinolones, second line injectables, ethionamide, cycloserine, and in some patients PAS is performed. The treatment follows up sputum cultures are performed on LJ media and are subject of pDST in case of persistent culture positivity or on clinician demand.

For the study purpose the regrown of the stored isolates was performed on LJ media, no additional pDST were done. The pDST results from initial sputum samples (not additionally tested on regrown isolates) tested on admission or during the routine follow up was considered.

Microbiological tests for Mtbc detection (microscopy, GeneXpert Mtbc/RIF (Ceiphield), BACTEC MGIT 960 (Becton Dickinson, USA) and solid media cultures) were performed according to manufactures recommendations and as instructed in the WHO guidelines. As well phenotypic antimicrobial susceptibility testing was performed using WHO recommended critical concentrations and according to the WHO guidelines.

Phenotypic antimicrobial susceptibility testing

MGIT cultures were incubated for a total of 6 weeks before being reported as negative. The purity of the MGIT culture was checked by Ziehl–Neelsen stain, blood agar plate, and MPT64 antigen rapid test (BD Microbiology Systems, USA). For the preparation of the inoculation procedure, mycobacterial suspensions were used undiluted from day 1 to 2 following positivity, while the suspensions were diluted 1:5 with sterile saline from day 3 to 5. Susceptibility testing using the BACTEC MGIT 960 system was performed with the following final drug concentrations: 1.0 µg/ml for SM, 0.1 IHN, 1.0 µg/ml for RMP, and 5.0 µg/ml for EMB. DST results were determined at the time the growth control tube displays >400 Growth Units (GU) between day 4 and day 13. The interpretation of the DST results was as follows: no growth in the drug vial "susceptible," 1–399 GU "intermediate," and >400 GU "resistant."

Critical concentrations for LJ DST testing

Approximately 0.1 mL of the specimen was inoculated onto the LJ slant and were incubated at 37°C. The LJ tubes were examined weekly for 8 weeks before discarding as negative. The positive LJ cultures were used for critical concentrations for LJ DST. The anti-TB agents recommended for DST using LJ media and the established critical concentrations for testing are listed: Isoniazid 0.2 mg/L, Rifampin 40.0 mg/L, Ethambutol 2.0 mg/L, Streptomycin 4.0mg/L. The Results as "susceptible" or "resistant" were reported after 3 weeks if there is growth on the medium containing the anti-TB agent and all other parameters are satisfactory.

Isolation of genomic DNA

M. tuberculosis complex strains were inoculated on Löwenstein Jensen medium at 37°C, until was good growth (abundant). Colonies were transferred to a microcentrifuge tube (2.0 ml) containing 400 µl TE buffer and heated for 20 min at 80°C to kill the bacteria. After 3 min centrifugation at 13,000 g we discarded the supernatant and added 400 µl TE-buffer, followed by vortexing to separate cells. We then added 50 µl lysozyme (10 mg/ml) vortexed briefly and incubated the solution overnight at 37°C. The next day, we added 70 µl 10% SDS, 5 µl proteinase K (10 mg/ml), vortexed softly and incubated the solution 10 min at 65°C. Subsequently, we added 100 µl 5M NaCl, 100 µl CTAB/NaCl (pre-warmed at 65°C), followed by vortexing and incubation for 10 min at 65°C. we then added 750 µl Chloroform/Isoamyalcohol mix (24:1), inverted the tube few times and centrifuged at room

temperature for 15 min at 13,000 g. The aqueous supernatant was carefully transferred to a new microcentrifuge tube, and 0.6 volume isopropanol was added to precipitate the nucleic acids for 30 min at −20°C (or longer). We then centrifuged for 10 min at room temperature at 13,000 g, discarded the supernatant and washed the DNA in 0.5 ml of cold 75% Ethanol while inverting the tube few times, followed by 5 min centrifugation at room temperature and discarding the supernatant cautiously. The DNA-pellet was dried at 60°C for ≈10 min, and DNA was eventually dissolved in 100 µl TE-buffer at 37°C for 30 min or at room temperature until DNA was completely dissolved.

Whole genome sequencing, and genotypic characterization

WGS was performed using Illumina technology (NextSeq 500, and Nextera XT library preparation kit) according to the manufacturer's instructions (Illumina, San Diego, CA, USA). Raw read data (ENA study accession number PRJEB58814) were mapped to the *M. tuberculosis* H37Rv genome (NC_000962.3) using MTBseq (*1*) and aiming for a minimum genome wide coverage of 50x. Variants including single nucleotide polymorphisms (SNPs) and insertions and deletions (InDels) were called with the following thresholds: minimum coverage of four reads in both forward and reverse orientation, four reads calling the allele with at least a phred score of 20, and an allele frequency of 75%. For a concatenated SNP alignment, we considered genome positions that fulfilled these thresholds in at least 95% of all isolates. We further excluded mutations in drug resistance associated genes and repetitive regions, and when >1 mutation occurred in a 12bp window to avoid possible homoplasy effects and putative recombination artifacts.

Genotypic drug resistance was inferred on the basis of a curated mutation catalog (*2*) used at the Supranational Reference Laboratory (SRL), Research Center Borstel, Germany, based on information available on 2020–05–10. Mutations implicated in drug resistance were called with a minimum coverage of 2 reads in both forward and reverse orientation, and two reads calling the allele with at least a phred score of 20. The phylogenetic lineage was determined using a SNP barcode (*3*).

Re-infection of the focus patients with an MDR/RR strain was confirmed by a pairwise genetic distance measurement exceeding 12 SNPs between the non-MDR/RR isolate and the

consecutive MDR/RR isolate. The MDR/RR isolate was further subjected to a transmission analysis to identify putative index cases.

Inference of patient-to-patient transmission

We used a maximum pairwise distance between any two isolates of 5 SNPs to identify molecular clusters as surrogate marker for recent transmission (*4*). A possible direct transmission between the admitted MDR/RR-TB patients and the focus patients were inferred when both isolates belonged to the same molecular cluster and shared identical genotypic drug resistance profiles. To visualize Mtbc isolates from patients who possibly acquired MDR/RR-TB in the hospital, we performed a core genome multi locus sequence type (cgMLST) analysis as described previously (*5*) with SeqSphere v8.4 classifying cgMLST clusters based on <1 allele pairwise distance between any two isolates. Further details are provided in the Supplemental Materials Methods file.

After completion of the follow-up, stored isolates from sputum cultures of patients with MDR/RR-TB at baseline and of patients with drug susceptible TB at baseline and MDR/RR-TB during follow-up were regrown and Mtbc DNA was extracted (details on DNA isolation are presented in Supplementary method file). Whole genome sequencing of the extracted Mtbc DNA was performed following transfer of the DNA to the Research Center Borstel, Germany. WGS data were used to possibly identify the potential source of the nosocomial transmission of the MDR/RR Mtbc strains.

Statistical analysis

Continuous variables were expressed as the mean or median with standard deviation (SD) or inter quartile range (IQR) correspondingly, while categorical variables were presented as frequencies. For the comparisons of means and medians Student's t-test or Mann-Whitney test were applied. For the comparison of frequencies, the exact Fisher test was used. A p-value of <0.05 was considered significant.

Results

General cohort characteristics

A total of 2,627 admissions were recorded at the Chiril Draganiuc Phthisiopneumology Institute during the study period. One hundred twenty-three patients had more than one

admission (109 patients had 2 admissions, 13 had 3 admissions and 1 had 4 admissions), amounting to 2,490 individual patient admissions during the study period. The mean age was 50.9 SD±17.4 years; 1623 (65.2%) were male. [is it of interest to add two sentences of differences between the wards?] Demographic and epidemiologic characteristics of the patients admitted to different hospital wards are presented in Table 1, main article.

Diagnosis of tuberculosis and *M. tuberculosis* complex drug resistance

During the hospital admission 55.4% (1379/2490) of the patients were diagnosed with tuberculosis (Appendix 1 Figure 1). Culture confirmation of TB was possible in 938 (68.0%) cases. In addition, Xpert MTB/RIF identified Mtbc DNA in 78/441 (17.7%) of patients with negative Mtbc cultures. Thus, the total number of patients with a confirmed diagnosis of TB by culture or Xpert MTB/RIF was 1016/1379 (73.7%). In 363/1379 (26.3%) of the TB patients all microbiological tests for Mtbc were negative, and the diagnosis of TB was made clinically.

Drug-susceptible strains of Mtbc were found in 567/938 (60.5%), mono/polydrug-resistant Mtbc in 64/938 (6.8%) and MDR/RR Mtbc in 307/938 (32.7%) of patients with detectable Mtbc in culture. 297/307 (96.7%) MDR/RR strains were available for analysis (20/307 were admitted before the study period and *Mtb*c had already become undetectable by culture and therefore, after study launch, they were excluded as positive specimens were outside study start and end dates).

In hospital movement

406/2627 (15.4%) of initial hospital admissions were to the "Phthisiopneumology" Department (Dept) #1, 459/2627 (17.5%) to the "Phthisiopneumology" Dept. #2, 1219/2627 (46.4%) to the "Phthisiopneumology" Dept. #3, 167/2627 (6.4%) to the MDR-TB Dept. and 376/2627 (14.3%) to the Dept. of extrapulmonary TB (epTB) and Surgery.

The median length of hospital stay was 22 [IQR 9–62] days. Patients in the MDR-TB Dept. had the longest stay (median 129 [IQR 93–168] days while patients admitted to the Dept. #3 had the shortest stay (9 days [IQR 8–13.5]) (Appendix 1 Figure 2, panel A).

After admission 75 of the patients were transferred to a different department than that where they were initially hospitalized. One of the transferred patients was transferred twice and another one was transferred three times among different hospital departments. Twenty-four patients initially admitted to Dept. #1 or Dept. #2 were later transferred to the MDR-TB Dept.

and other 4 patients to Dept. #3. Nine of the patients initially admitted to Dept. #3 (a non-TB department) were later transferred to Dept. #1 or Dept. #2 (TB/non-MDR-TB departments) and other 4 patients to the MDR-TB Dept. Three patients initially admitted to MDR-TB Dept. (with MDR tuberculosis) were later transferred to Dept. #2 (1 patient), Dept. #3 (1 patient) and Dept. epTB & Surgery (1 patient). Several patients initially admitted to different Depts. (Dept. #1: 5 patients, Dept. #2: 4 patients, Dept. #3: 7 patients, MDR-TB Dept.: 1 patient) were transferred to the epTB and Surgery Dept. Thirteen patients initially admitted to the epTB & Surgery Dept. were transferred to different non-surgical departments (Appendix 1 Figure 2, panel B). The transfer rate from different hospital departments was comparable among them (5.9% from Dept. #1, 3.3% from Dept. #2, 1.6% from Dept. #3, 1.8% from MDR-TB Dept. and 3.5% from the epTB & Surgery Dept.).

The median length of stay till the transfer to another department was 7 [IQR 4–19] days, the shortest being in patients from MDR-TB Dept. Three [IQR 1–75] days and the longest in those from Dept. #2 and the epTB & Surgery Dept. Thirteen [5.-32] days and 12 [4–48] correspondingly. The median length of stay till the transfer from Dept. #1 was 6 [2–7.5] days and from Dept. #3 9 [4–13.5] days, respectively (Appendix 1 Figure 2, panel C).

Forty-one patients with MDR/RR-TB were initially admitted to one of the non-MDR-TB departments. Of them 33 patients were later transferred to MDR-TB Dept. and 8 patients to a different non MDR-TB Dept. The 33 patients transferred to MDR-TB Dept. were initially admitted to Dept. #1 (n = 13), Dept. #2 (n = 10), Dept. #3 (n = 3) and epTB & Surgery Dept. (n = 7) while those 8 transferred to a non MDR-TB Dept. were initially admitted to Dept. #1 (n = 3), Dept. # 3 (n = 2) and epTB & Surgery (n = 3) (Appendix 1 Figure 3, panel A). The median and cumulative duration of stay of patients with MDR/RR-TB on non-MDR/RR-TB departments were 7 days [IQR 4–18] and 631 days respectively (Appendix 1 Figure 3, panel B). The median and cumulative number of sharing-room contacts of patients with MDR/RR-TB on non-MDR/RR-TB wards were 3 [IQR 2–5] and 144 patients, respectively.

MDR/RR-TB during follow up

We found 17 patients ('focus patients') with drug-susceptible Mtbc strains on enrollment and a re-infection with an MDR/RR-Mtbc strain on follow up. A re-infection was confirmed by a genetic distance between the two isolates of more than 12 SNPs. The median time from the study

enrollment till isolation of the MDR/RR-Mtbc strains from these 17 patients was 215 [IQR 48–301] days. Only one of the 144 roommates of the 41 patients with MDR/RR-TB initially admitted to a non-MDR-TB ward were in the same room with one of the 17 focus patients who potentially acquired MDR/RR-TB on the non-MDR-TB ward. Consequently, we investigated molecular clusters and antibiotic resistance profiles to identify putative MDR-TB contacts. We further tracked overlapping stays in the hospital, department, and rooms until the focus patients were diagnoses with MDR/RR-TB.

Molecular epidemiologic analysis

For the 297 patients with culture positive MDR/RR-TB at study enrollment and the 17 focus patients with an MDR/RR-Mtbc re-infection during follow-up, 274 next generating sequencing datasets could be generated (40 MDR/RR-Mtbc could not be regrown from the biobank). For 6 Mtbc isolates, the DNA isolate was contaminated with DNA from other mycobacteria than Mtbc and these strains were excluded from further analysis, leaving 268 datasets for the molecular epidemiologic analysis including all 17 strains from the patients who were identified with MDR/RR-TB during follow up.

The bacterial population comprised Mtbc lineage 2 isolates (116/268, 43.3%) and lineage 4 isolates (152/268, 56.7%). We identified among lineage 2 isolates previously described clades, which have been found in many high MDR-TB incidence settings in Eastern Europe, the Russian Federation and Central Asia (*1*): isolates typed as Central Asia L2-sublineage (67/116, 57.8%), Central Asia outbreak (21/116, 18.1%) and Europe/Russia W148 outbreak (25/116, 21.6%) were dominating among lineage 2, while lineage 4 almost exclusively comprised sub-lineage 4.2.1, URAL genotype (139/152, 91.4%) (Figure 1).

We used the sequence data to predict the resistance phenotype based on direct association with previously described resistance conferring SNPs [11]. In the whole MDR/RR-TB cohort, drug resistance rates were high for streptomycin (247/268, 92.2%), ethambutol (165/268, 61.6%), pyrazinamide (132/268, 49.3%), prothionamide (234/268, 87.3%) and kanamycin (Appendix 1 Figure 5). We found mutations, which confer resistance against moxifloxacin and levofloxacin in 68/268 (25.4%), for amikacin in 35/268 13.1%), for capreomycin in 26/268 (9.7%), for PAS in 61/268 (22.8%), and for D-cycloserine in 16/268 (6.0%) of the isolates.

To point out putative transmission events between the focus patients and concurrently admitted MDR-TB patients, we performed a molecular cluster analysis based on pairwise genetic distance between all isolates (see methods). One patient (sample ID 24) was placed in the largest single cluster including 53 lineage 4.2.1 (URAL, cluster 26) isolates initially assuming an extensive ongoing nosocomial transmission scenario (Figure 2). However, considering the low genetic diversity of the dominating lineage 4.2.1 (URAL) clade in the phylogeny (Figure 1), we have to assume that these strains are highly prevalent in the community in the Republic of Moldova. Furthermore, this patient (sample ID 24) did not have any identified hospital stay overlap with other MDR-TB patients in our cohort. Overall, 124/268 (46.3%) patients were part of one of the 28 identified clusters, including 7/17 focus patients (Appendix 2). Two patients (sample ID 28 and 37) were re-infected with the same pre-XDR strain (cluster 28) harboring identical resistance mediating mutations but lacking any hospital overlaps with each other or any other MDR-TB patient during their time on the non MDR-TB ward (Appendix 2). Likewise, another possible nosocomial re-infection event (cluster 27) involving a focus patient (sample ID 30) who was infected with the same strain (0 SNP difference) as another admitted MDR-TB patient was not plausible due to the absence of overlapping admission times (Appendix 2). In cluster 12 the focus patient (sample ID 12; MDR) overlapped for 4 days in the hospital with a pre-XDR patient, however, additional mutations conferring resistance against pyrazinamide and moxifloxacin in the putative index patient also excluded a direct link. Thus, only 2/17 focus patients (sample ID 14 and ID 285) had a possible direct link in the hospital with less than 6 SNP difference between the infecting Mtbc strains as well as 13 and 29 days overlap in the hospital with their putative index case (Table 2). For the other re-infection events, we need to assume a contact outside the hospital, an undetected mixed infection at baseline, or a missing culture from our cohort.

Patients NOR 297 (male, 41 y.o.) and NOR 133 (male, 55 y.o.) had a baseline grade of sputum smear microscopy 1+ and 2+, respectively. The baseline time to culture positivity (TTP) in NOR 297 was 30 days (tested on LJ) and in NOR 133 - 14 days (tested on MGIT). In 244/307 culture proven MDR-TB patients initial MDR strains was isolated on MGIT while in other 63 on LJ media. The median age in all patients with MDR/RR-TB was 38 years (IQR 28–48), 75.6% of them were men. The median baseline sputum smear grade in patients inappropriately located initially on a non-MDR/RR-TB department and of patients with MDR/RR-TB who were located

on an MDR-TB ward from the beginning was similar 1–9 AFB in 100 fields (IQR 0 −1+ grade). The baseline time to culture positivity, in MDR-TB patients admitted to the appropriate ward depended of the used media and was 15 days (IQR 12–20, n = 37) on MGIT and 55 days (IQR 52–55.5, n = 4) on LJ, while in those with initial admission to a non-appropriate ward was 16 days (IQR 11–23, n = 207) on MGIT and 38 days (29–50, n = 59) on LJ (Appendix 1 Table). None of the differences were statistically significant.

References

1. Bantubani N, Kabera G, Connolly C, Rustomjee R, Reddy T, Cohen T, et al. High rates of potentially infectious tuberculosis and multidrug-resistant tuberculosis (MDR-TB) among hospital inpatients in KwaZulu Natal, South Africa indicate risk of nosocomial transmission. PLoS One. 2014;9:e90868. PubMed https://doi.org/10.1371/journal.pone.0090868

2. Gandhi NR, Weissman D, Moodley P, Ramathal M, Elson I, Kreiswirth BN, et al. Nosocomial transmission of extensively drug-resistant tuberculosis in a rural hospital in South Africa. J Infect Dis. 2013;207:9–17. PubMed https://doi.org/10.1093/infdis/jis631

3. Smith JP, Modongo C, Moonan PK, Dima M, Matsiri O, Fane O, et al. Tuberculosis attributed to transmission within healthcare facilities, Botswana—The Kopanyo Study. Infect Control Hosp Epidemiol. 2022;43:1603–9. PubMed https://doi.org/10.1017/ice.2021.517

4. Fox GJ, Redwood L, Chang V, Ho J. The effectiveness of individual and environmental infection control measures in reducing the transmission of Mycobacterium tuberculosis: a systematic review. Clin Infect Dis. 2021;72:15–26. PubMed

5. Yang C, Sobkowiak B, Naidu V, Codreanu A, Ciobanu N, Gunasekera KS, et al. Phylogeography and transmission of M. tuberculosis in Moldova: a prospective genomic analysis. PLoS Med. 2022;19:e1003933. PubMed https://doi.org/10.1371/journal.pmed.1003933

Appendix 1 Table. Characteristics of patients with MDR/RR-TB inappropriately located initially on a non-MDR/RR-TB ward and of patients with MDR/RR-TB who were located on an MDR-TB ward from the beginning

Characteristic	MDR/RR-TB patients admitted initially to an appropriate ward n = 266	MDR/RR-TB patients admitted initially to an inappropriate ward n = 41
Age, median (IQR)	38 (29–48)	38 (27–50)
Sex, male (%)	74.1	85.4
Smear microscopy grade	1–9/100 (0–1+)	1–9/100 (0–1+)
Time to culture positivity on MGIT (days)	16 (11–23)*	15 (12–20)†
Time to culture positivity on LJ (days)	38 (29–50)‡	55 (52–52.5)§
Extrapulmonary involvement additionally to pulmonary lesions (%)	7.9	12.2

*n = 207.
†n = 37.
‡n = 59.
§n = 4; none of the differences were statistically significant.

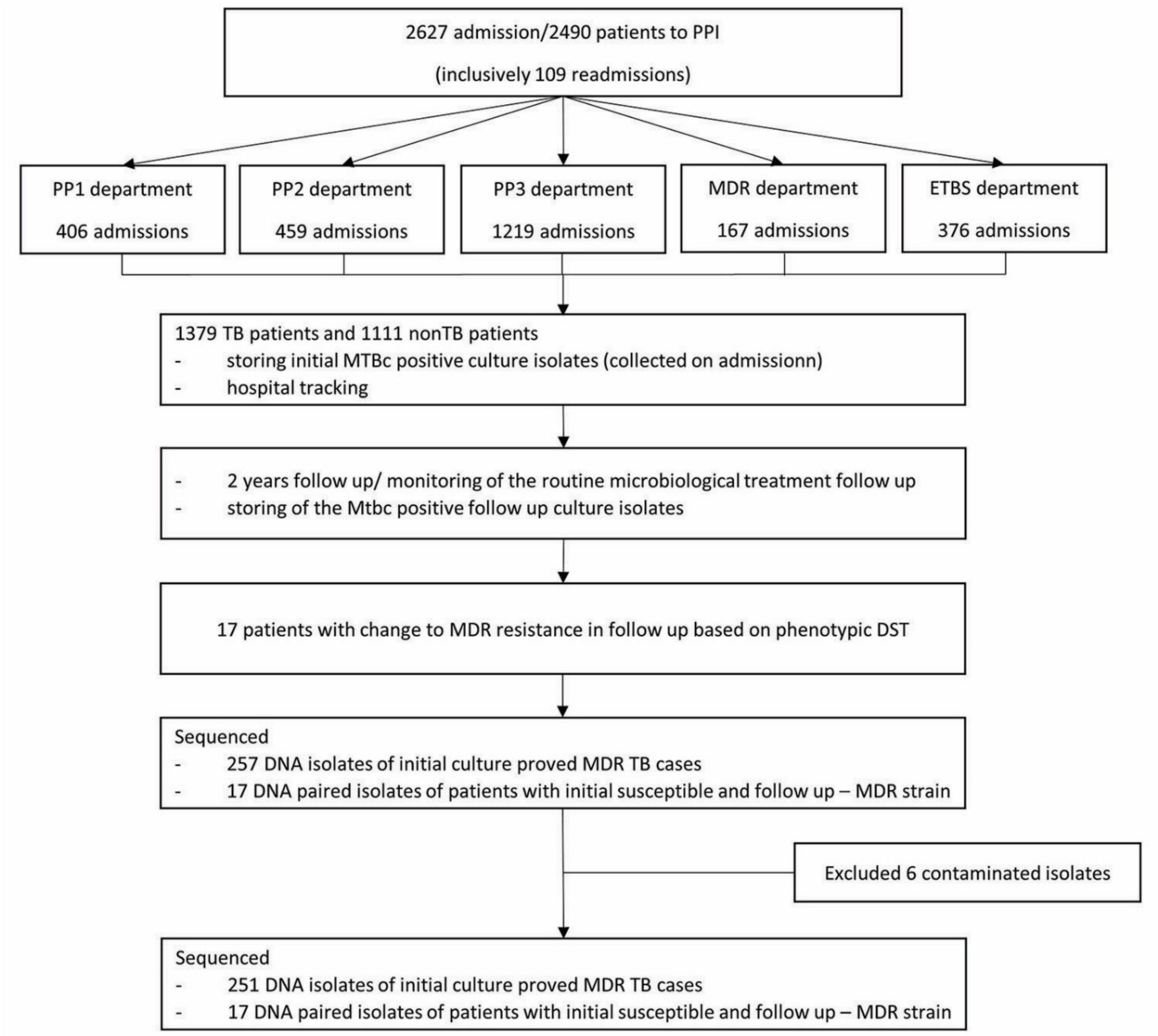

Appendix 1 Figure 1. Study flowchart

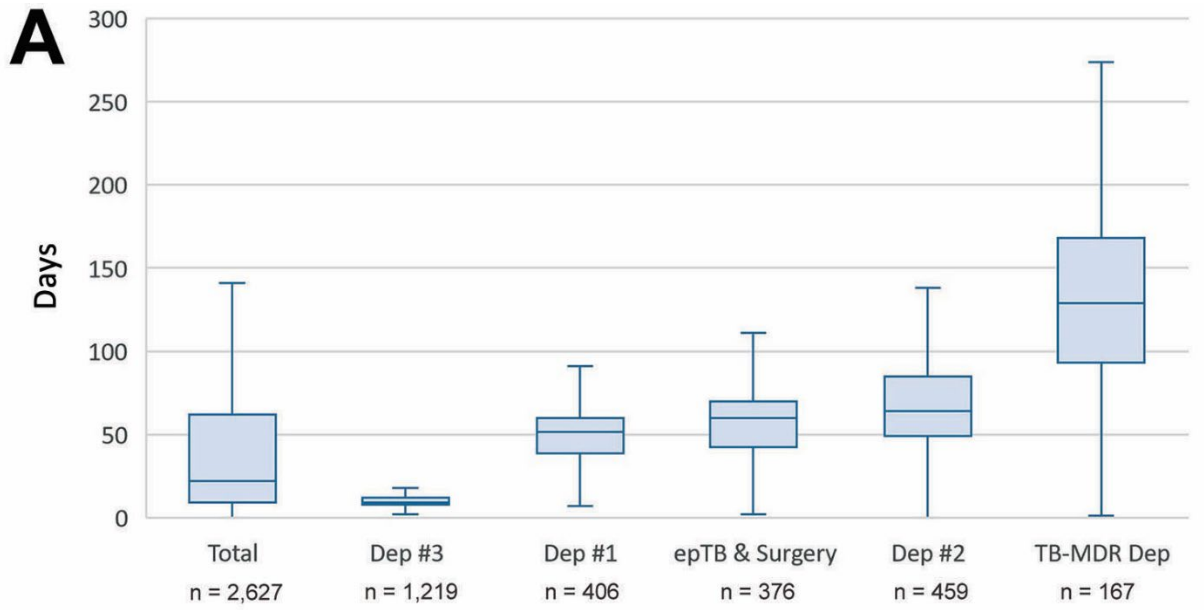
A
Days
300
250
200
150
100
50
0
Total
n = 2,627
Dep #3
n = 1,219
Dep #1
n = 406
epTB & Surgery
n = 376
Dep #2
n = 459
TB-MDR Dep
n = 167

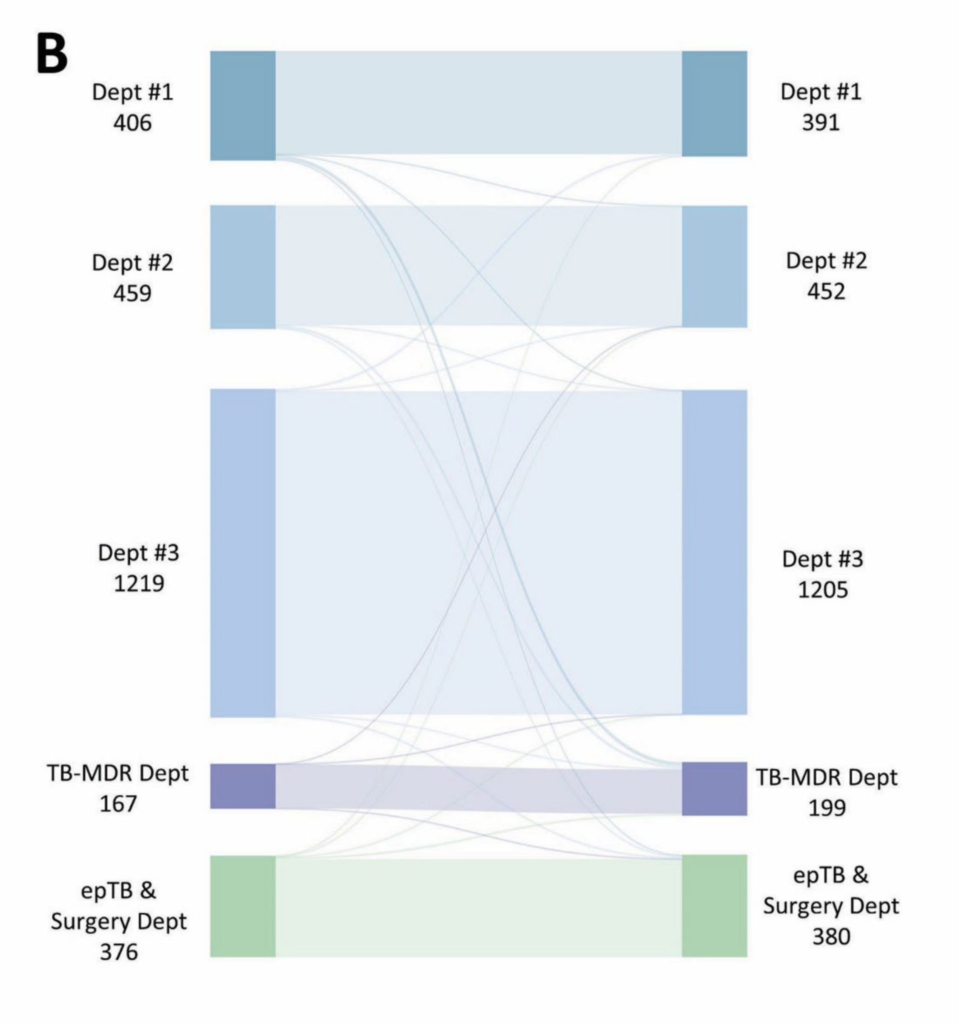
B
Dept #1
406
Dept #2
459
Dept #3
1219
TB-MDR Dept
167
epTB &
Surgery Dept
376
Dept #1
391
Dept #2
452
Dept #3
1205
TB-MDR Dept
199
epTB &
Surgery Dept
380

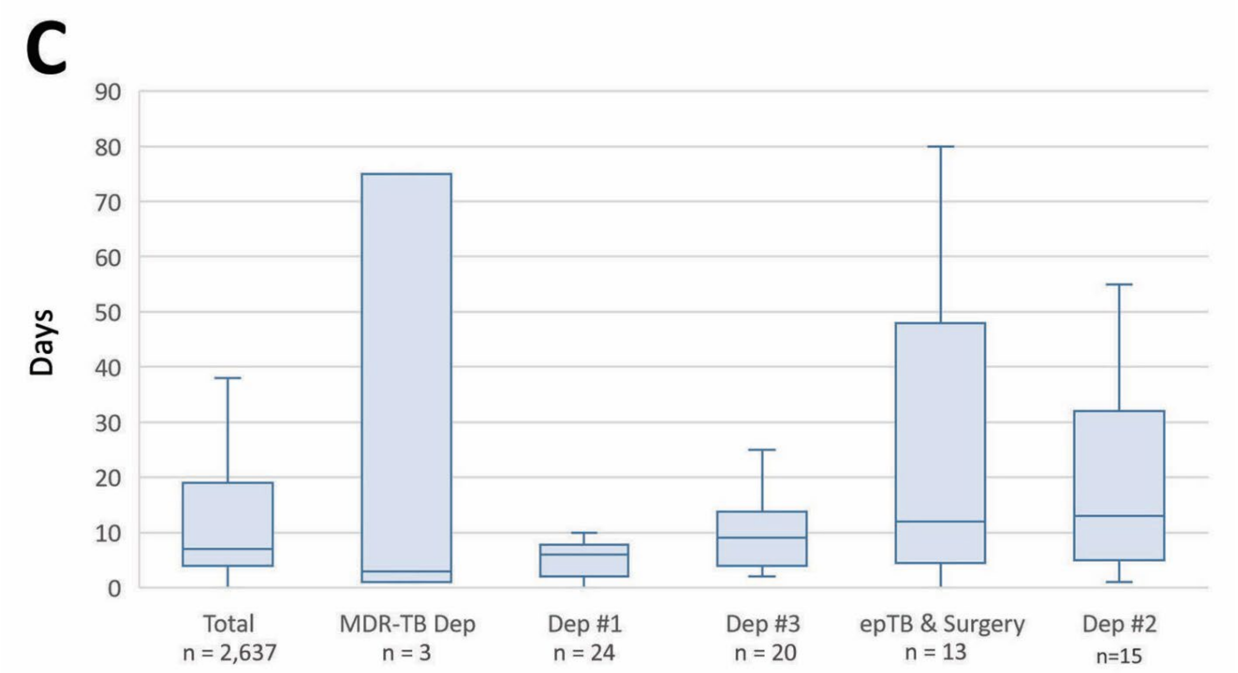

Appendix 1 Figure 2. A. Duration of hospital stay and transfers by hospital departments. **B.** Transfers to other department after initial admission, by initial department of admission (75 transfers out of 2627 admission). **C.** Duration of department stay till the transfer (days), by departments. Dept. = Department; TB = tuberculosis; MDR-TB = multidrug-resistant tuberculosis, ep-TB = extrapulmonary TB

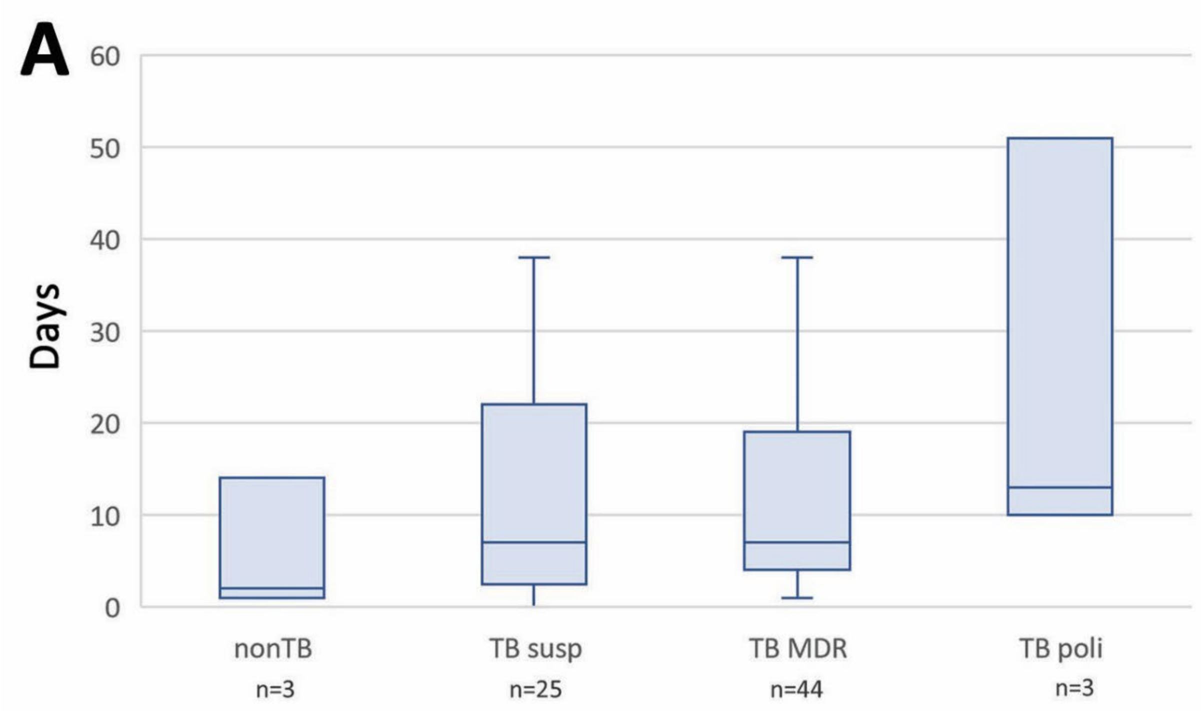

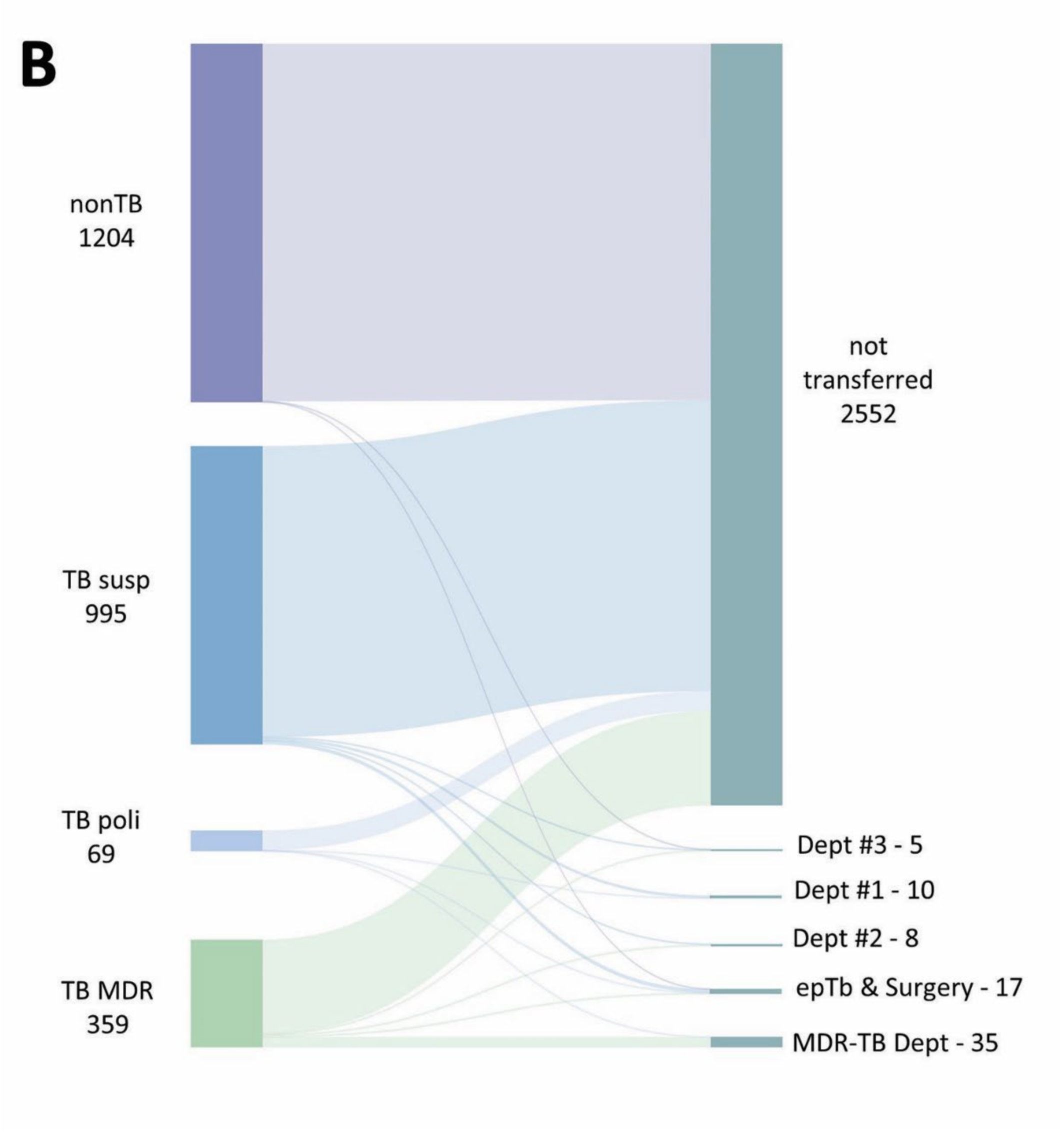

Appendix 1 Figure 3. A: Duration of the hospital stay and department transfers by resistance pattern of *M. tuberculosis* complex. **B:** Transfers to other department after initial admission, by resistance (75 transfers out of 2627 admission). Dpt. = Department; TB = tuberculosis; MDR-TB = multidrug-resistant tuberculosis, ep-TB = extrapulmonary TB, TB susp = susceptible tuberculosis, TB poli = polyresistant tuberculosis, nonTB = pulmonary diseases other than tuberculosis.

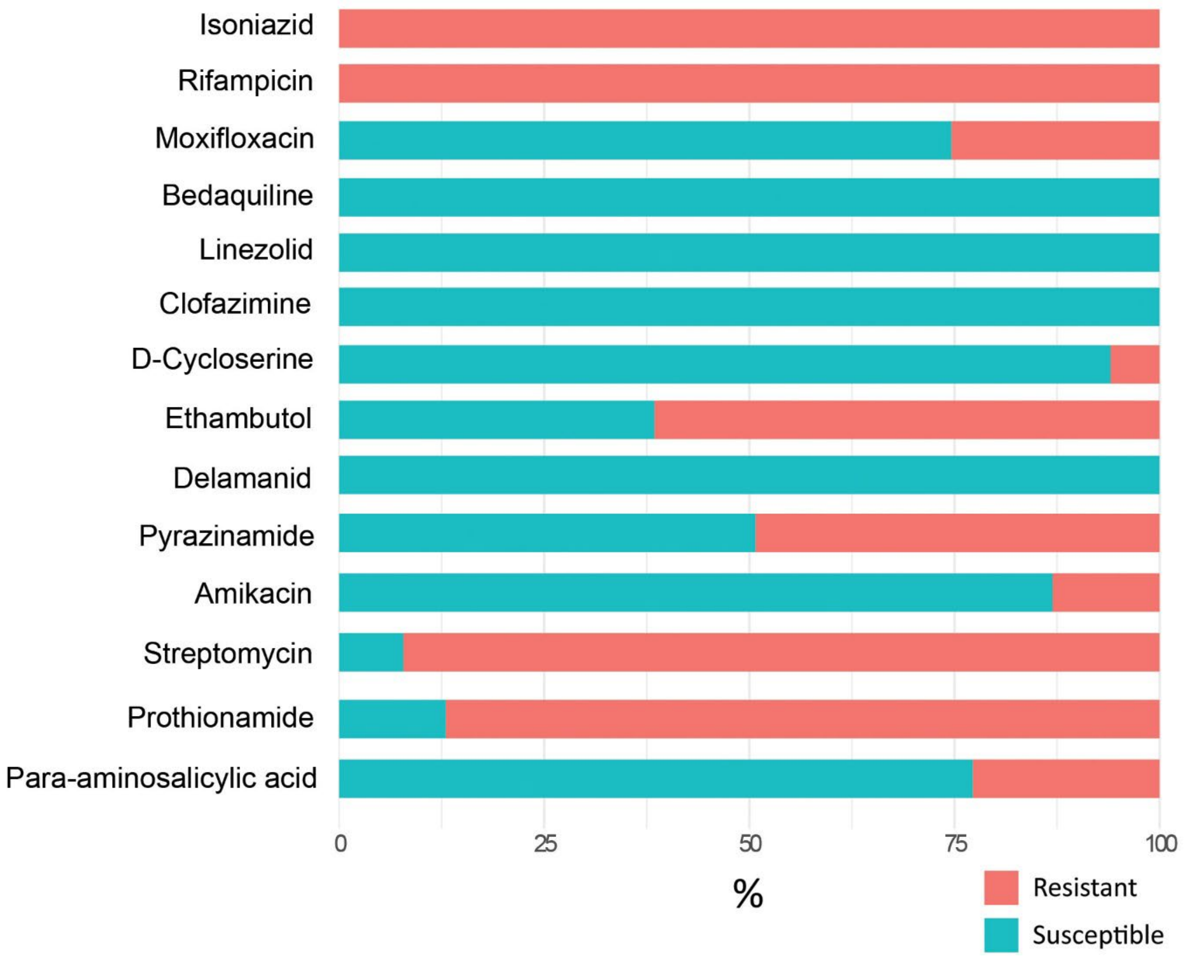

Appendix 1 Figure 4. Genotypic drug resistance proportions among 268 MDR-Mtbc isolates collected between 2014–2015 at the Phthisiopneumology Institute in the Republic of Moldova.
